# Characterization of *Bacillus velezensis* RDA1 as a Biological Control Agent against White Root Rot Disease Caused by *Rosellinia necatrix*

**DOI:** 10.3390/plants11192486

**Published:** 2022-09-22

**Authors:** Shailesh S. Sawant, Janghoon Song, Ho-Jin Seo

**Affiliations:** Pear Research Institute, National Institute of Horticultural & Herbal Science, Naju 58216, Korea

**Keywords:** *Rosellinia necatrix*, biocontrol, *Bacillus velezensis*, white root rot

## Abstract

White root rot disease, caused by *Rosellinia necatrix*, poses a threat to several tree crops; hence, effective and sustainable strategies to control this disease remain warranted. This study identified an effective *R. necatrix* biocontrol agent by isolating 32 strains from soil samples collected from white root rot-infested organic pear orchards, among which RDA1 exhibited the most potent growth-inhibitory effects. Microbiological and 16S rRNA gene sequencing analyses revealed that the bacterial isolate belonged to the *Bacillus* genus and exhibited 100% nucleotide sequence similarity with *Bacillus velezensis* species in the GenBank. This strain showed strong antifungal activity against four *Rosellinia necatrix* strains and harbored genes essential for lipopeptide, polyketide, and tripeptide bacilysin biosynthesis. RDA1 produced volatile compounds that suppressed the development of phytopathogens and possessed plant growth-promoting traits, such as phosphate solubilization, and indole-3-acetic acid and siderophore production. *B. velezensis* RDA1 has a significant potential application in sustainable agriculture and can be used to suppress white root rot disease infections and to improve plant growth.

## 1. Introduction

The causative agent of white root rot, *Rosellinia necatrix* Berl. Ex. Prill., is a necrotrophic phytopathogenic fungus that affects several important crops such as pears, cotton, avocados, nuts, apples, citruses, cherries, and mangoes [1,2,3]. Its host range, comprising more than 350 plant species, has been listed by the USDA-ARS (Fungal Databases, U.S. National Fungus Collections (ars-grin.gov)), which is regularly updated as new hosts are discovered. *R. necatrix* infects both the surfaces of roots and tissues under the bark, thereby damaging the established orchards and causing severe losses in the nursery [2]. White root rot disease affects fruit trees and causes canopy decline, leaf drop, wilting, and eventually death of the infected tree, it remains a major agricultural challenge worldwide [4]. Additionally, *R. necatrix* can survive for many years by thriving in the residues of susceptible crops [5] and by spreading through mycelial strand growth or direct root-to-root contact between infected and healthy plants [6]. Controlling the white root rot disease is difficult since *R. necatrix* can tolerate drought conditions and a wide range of soil pH, has a wide host range, and is resistant to common fungicides [7]. Multiple strategies have been applied to control *R. necatrix*, such as cultural control, soil disinfestation, and solarization [2]. For instance, in orchards, chemicals such as chloropicrin, carbendazim, fluazinam, benzimidazole, and carbendazim are used to control this pathogen [1,3,8,9]. Although these control measures have been used in most farms, they remain ineffective, while annual repetitive supplementary planting is currently the only solution to controlling *R. necatrix*.

In addition, there are environmental concerns regarding chemical control strategies and the associated accumulation of toxic residues in the soil. Consequently, strategies that employ biological control agents (BCAs) may be an effective alternative to reducing the impact and the spread of the white root rot disease.

The application of plant growth-promoting bacteria (PGPB) as a BCA is promising for the management of numerous fungal diseases [4], as it promotes plant growth through various mechanisms, such as phytohormone (auxins, cytokinins, and gibberellins) production, siderophore production, phosphate solubilization, and nitrogen fixation. *Bacillus* species fungal antagonists PGPB [10]. As an important member of the *Bacillus* genus, *Bacillus velezensis* has been described as a heterotypic synonym for *B. amyloliquefaciens* [11]. Numerous strains of *B. velezensis* have been isolated and characterized for their ability to stimulate plant growth and production of antifungal metabolites [12,13]. These antagonistic bacteria produce several secondary metabolites involved in phytopathogens regulation [14]. Owing to these properties, certain *B. velezensis* strains have been used in agriculture as plant growth promoters and BCAs [4,15]. Other advantages of using *Bacillus* as BCAs include their resistance to unfavorable environmental conditions, the simplicity and low cost of setting up large-scale treatment, and their low contamination rate in dry-product formulation [16]. Furthermore, Mantell and Wheeler [17] have reported that the usage of antagonists or soil microorganisms is an ideal strategy to reduce *R. necatrix* colonization. Therefore, this study isolated a *B. velezensis* strain and evaluated its efficiency in controlling *R. necatrix*. We isolated an antagonistic bacterial strain of *Bacillus velezensis* from organic pear orchards infested with white root rot and evaluated its antifungal activity and PGP traits. Our study proposes a potential source for BCAs against white root rot and as a bio-fertilizer in sustainable agriculture.

## 2. Results

### 2.1. Isolation and Identification of Strain RDA1

**Figure 1:**
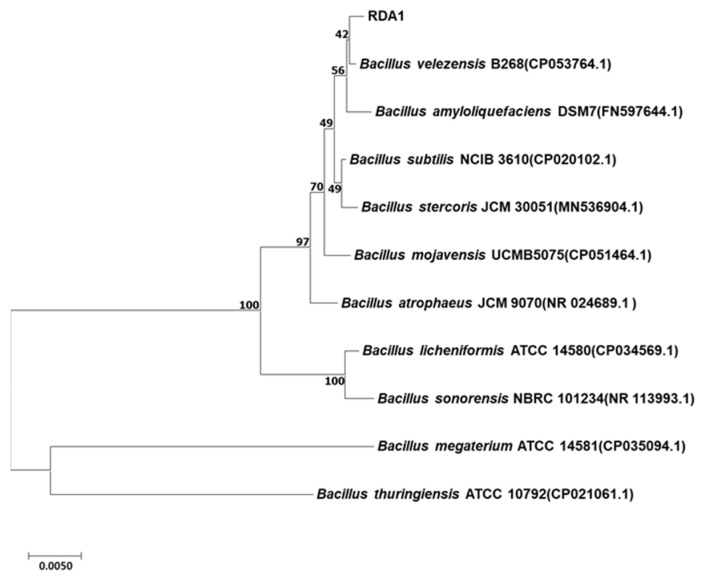
16S rDNA phylogenetic tree showing the relationship between the strain RDA1 and other *Bacillus* sp. strains. The tree was constructed using the neighbor-joining method via the MEGA X program. Kimura-2 parameter was used as the nucleotide substitution model. The bootstrap values (%) presented at the branches were calculated from 1000 replications. *Bacillus thuringiensis* ATCC 10792 (CP021061.1) was used as an out-group. The scale bar indicates 0.005 substitutions per nucleotide position.

In this study, more than 100 bacterial strains were isolated from the rhizosphere soil of healthy pear trees in orchards infested with white root rot disease, of which 32 isolates exhibited an antagonistic ability against *R. necatrix.* RDA1, the most potent isolate with more than 70–80% inhibitory activity against all strains of *R. necatrix* (KACC 40446, 40445, 40447, and 40168), was selected for further analysis. 16S rRNA gene sequencing analysis revealed that the isolated strain RDA1 belonged to the genus *Bacillus*. The results of the BLAST search revealed that the 16S rRNA gene sequence of RDA1 was highly similar to that of the *Bacillus velezensis* strain B268 (CP053764.1). The partial 16S rRNA nucleotide gene sequence of strain RDA1 was deposited into GenBank under the accession number ON159294. Furthermore, the phylogenetic tree constructed using the neighbor-joining method showed that the isolate RDA1 was most closely related to strain B268 of *Bacillus velezensis* (Figure 1).

### 2.2. In Vitro Antifungal Efficacy of RDA1 against R. necatrix

**Figure 2:**
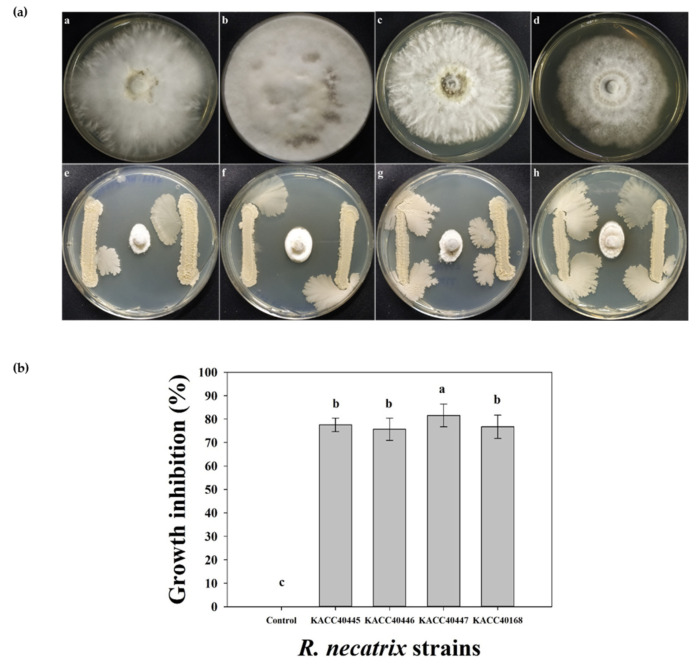
Antifungal activities of the isolated *Bacillus velezensis* RDA1 against four different strains of *R. necatrix* fungi KACC 40445, 40446, 40447, and 40168 in vitro. (**a**) A 5 mm fungus plug was inoculated into the center of the potato dextrose agar (PDA) medium and dual-culture plate assay. Plates a, b, c, and d are the controls for *R. necatrix* fungi KACC 40445, 40446, 40447, and 40168, respectively. Plates e, f, g, and h contain dual cultures of RDA1 and fungal pathogens. (**b**) Antifungal activities were measured based on the size of the zones of inhibition of the pathogenic fungi. Zones of inhibition were expressed as percentages. Vertical bars indicate ± standard deviation (*n* = 9). Different lowercase letters above each bar indicate significantly different means, as determined using Duncan’s multiple range tests.

The in vitro antagonistic efficacy of RDA1 against *R. necatrix* was assessed using direct confrontation assays (dual-culture plates). The inhibition zones of the pathogenic fungi were measured as percentages on PDA plates. RDA1 showed high inhibitory activity against *R. necatrix* (KACC 40168, 40445, 40446, and 40447) (Figure 2a). The highest level of fungal growth suppression was 81.57 ± 4.86% against KACC 40447, followed by 77.53 ± 2.88%, 76.72 ± 4.97%, and 75.67 ± 4.72% against KACC 40445, KACC 40168, and KACC 40446, respectively (Figure 2b). These strong antifungal activities may be attributed to certain diffusible compounds, such as antibiotics, against *R. necatrix*.

### 2.3. Antifungal Efficacy of the RDA1 Cell-Free Supernatant (CFS)

**Figure 3:**
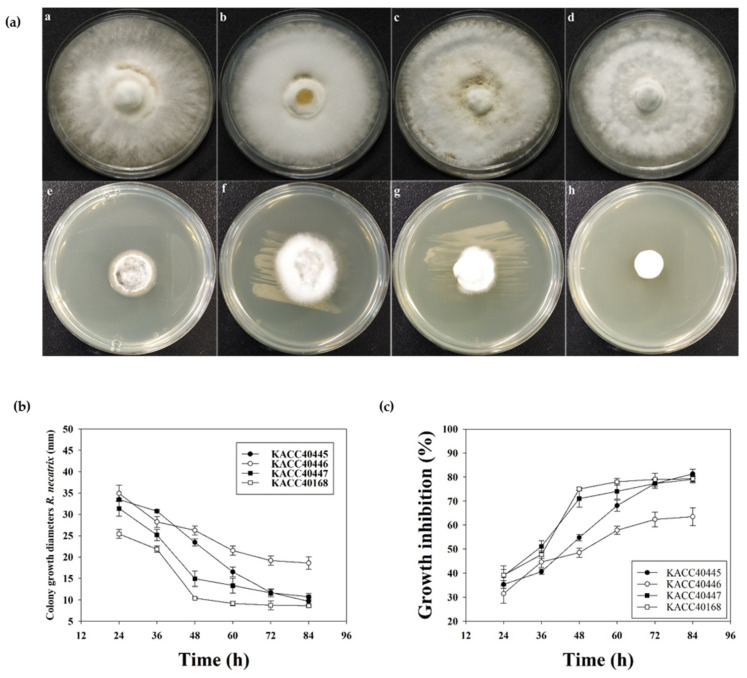
Effect of the cell-free supernatant (CFS) of RDA1 on the mycelial growth of different strains of *R. necatrix.* (**a**) Plates a, b, c, and d are the controls for *R. necatrix* strains KACC 40445, 40446, 40447, and 40168, respectively. Plates e, f, g, and h contain CFS collected at 90 h. (**b**) Effect of the CFS of RDA1 on colony growth diameter at various incubation time points (24, 36, 48, 60, 72, and 90 h). (**c**) Effect of the CFS of RDA1 on the growth inhibition percentage of *R. necatrix* at various incubation time points (24, 36, 48, 60, 72, and 90 h). Vertical bars indicate ± standard deviation (*n* = 9).

We next confirmed the release of antagonistic compounds in the growth medium by RDA1. To assess the effect of CFS of RDA1 on *R. necatrix* mycelial growth, CFS was prepared at 24, 36, 48, 60, 72, and 90 h, and Petri dish experiments were performed. Mycelial growth in (mm) and zones of inhibition of pathogenic fungi on PDA plates were determined as percentages. The CFS of RDA1 remarkably inhibited *R. necatrix* mycelial growth (Figure 3a). The 48 h CFS significantly inhibited the mycelial growth of KACC 40168 and 40447, and the 60 h CFS significantly inhibited that of KACC 40445 and 40446 (Figure 3b). The highest percentage of growth inhibition was observed following treatment with 90 h CFS in all the tested strains; 81.30 ± 1.92% was the highest growth inhibition percentage observed against KACC 40445, followed by 79.23 ± 1.16%, 79.09 ± 1.47%, and 63.49 ± 3.68% against KACC 40168, KACC 40447, and KACC 40446, respectively (Figure 3c). These results suggest that *B. velezensis* RDA1 secretes varying amounts of antimicrobial substances at different phases.

### 2.4. Antifungal Activity of Bacterial Volatile Compounds (VOCs) against R. necatrix

**Figure 4:**
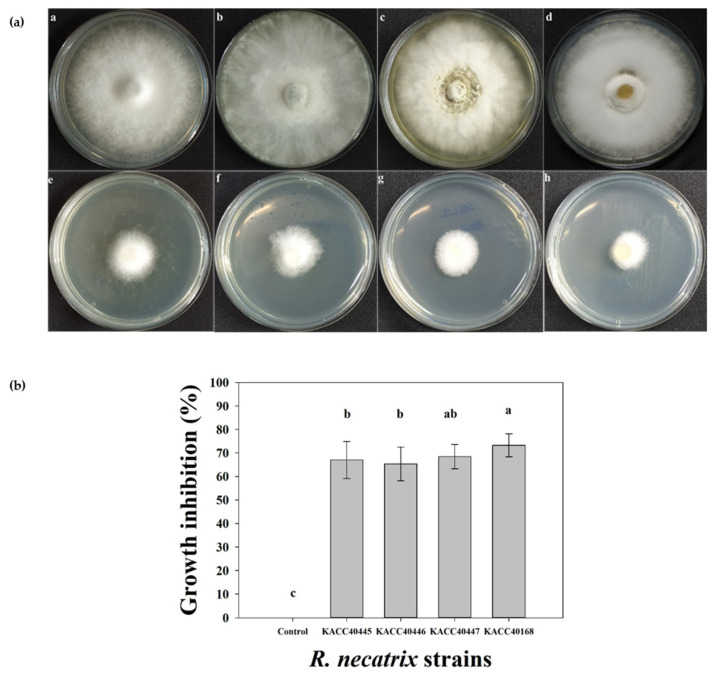
Effect of the volatile organic compounds (VOCs) produced by RDA1 on the mycelial growth of different strains of *R. necatrix.* (**a**) Plates a, b, c, and d are the controls for the *R. necatrix* strains KACC 40445, 40446, 40447, and 40168, respectively. Plates e, f, g, and h contain dual cultures of RDA1 and fungal pathogens. (**b**) The size of the zones of inhibition of the pathogenic fungi was used as a determinant of antifungal activities. The zone of inhibition was expressed as a percentage of the total area. Vertical bars indicate ± standard deviation (*n* = 9). Different lowercase letters above each bar indicate significantly different means, as determined via Duncan’s multiple range tests.

Microbial antagonist strains capable of producing volatile compounds (VOCs) that exhibit strong inhibitory activity against plant pathogens have received increasing attention [18]. Therefore, we assessed the antifungal ability of VOCs produced by RDA1 against *R. necatrix.* Two-sealed-base-plate assays were used to determine the antifungal activities of VOCs. Our results showed that VOCs produced by RDA1 inhibited the mycelial growth of *R. necatrix* compared with that of the control plates (Figure 4a). The highest fungal growth reduction was 73.25 ± 4.90% against KACC 40168, followed by 68.46 ± 5.18%, 67.03 ± 7.86%, and 65.33 ± 7.16% against KACC 40447, KACC 40445, and KACC 40446, respectively (Figure 4b). After 10 days of incubation, VOCs inhibited the growth of *R. necatrix* by approximately 60–70% compared with that of the control, suggesting that the bacterial VOCs had a significant inhibitory effect against fungal mycelia.

### 2.5. Plant Growth-Promoting Traits of RDA1

**Figure 5:**
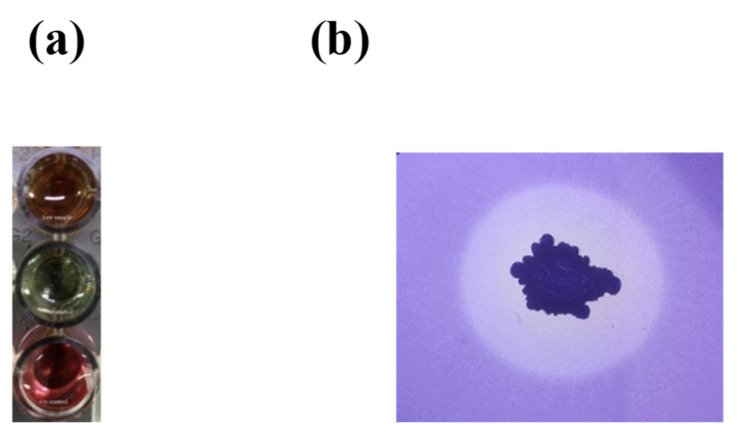
Plant growth-promoting traits of RDA1. (**a**) Positive indole acetic acid (IAA) production in the culture containing RDA1. The extreme lower well with pink color was used as positive control and the middle well was used as a negative control. IAA detection was indicated by a change in the color from yellow to pink in the test sample. (**b**) Phosphate solubilization assay. The growth of RDA1 on a NBRIP medium (a closer look at the clearing area surrounding the bacterial colony).

We evaluated several characteristics of RDA1 with potential association with plant growth promotion. These characteristics include the ability to produce indole-3-acetic acid (IAA) and siderophores and the capacity to solubilize phosphate. Both qualitative and quantitative tests were performed to identify the production of IAA in isolated RDA1. Changes in color from yellow to pink upon the addition of the Salkowski’s reagent to the cell free supernatant confirmed that strain RDA1 can produce IAA (Figure 5a). Furthermore, IAA was quantified by supplementing the strain with 2 mg mL^−1^ l-tryptophan in the culture medium; RDA1 produced 73.6 ± 4.9 μg mL^−1^ of IAA at a tryptophan concentration of 2 mg mL^−1^ in the culture medium. Additionally, RDA1 showed lower IAA content (23.2 ± 1.9 μg mL^−1^) without tryptophan supplementation (Table 1). These results suggest that the isolated strain RDA1 can potentially produce high levels of IAA when supplemented with exogenous tryptophan.

Siderophore production was assayed both qualitatively and quantitatively [19]. In the siderophore detection assay (CAS), changes in color from blue to orange indicates the production of siderophores by RDA. Additionally, RDA1 produced high levels of siderophores when cultured without Fe, and the total siderophore quantity (82.6 ± 4.3%) was reported as a percentage of siderophore units (PSU) in the culture medium without the addition of Fe(III) citrate. This result indicates that RDA1 can produce high levels of siderophores (Table 1).

The phosphate solubilization potential of RDA1 was assayed in a solid NBRIP medium [19]. In the NBRIP medium, Ca_3_(PO_4_)_2_ was used as the only source of inorganic phosphate. RDA1 could grow and solubilize inorganic phosphate, as shown by the clearing zone surrounding bacterial colonies, even after long incubation (Figure 5b).

### 2.6. Detection of Antibiotic Biosynthesis Genes

**Figure 6:**
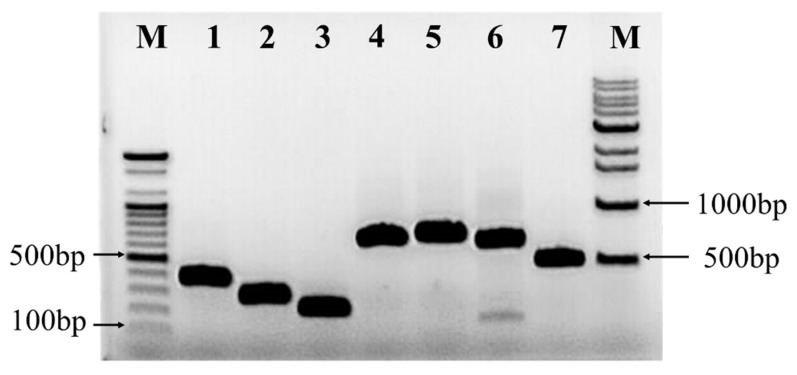
Agarose gel-electrophoresis of polymerase chain reaction fragments of antibiotic biosynthesis genes isolated from *B. velezensis* RDA1: Lane M, right side 100 bp and left side 1 kb DNA, Ladder from Bioneer; Lane 1, Bacillomycin gene *bmyB*; Lane2, Fengycin gene *fenD*; Lane 3, Surfactin gene *srfAA*; Lane 4, Macrolactin gene *mlnA*; Lane 5, Bacillaene gene *baeA*; Lane 6, Difficidin gene *done*; Lane 7, Bacilysin gene *bacA*.

The presence of genes involved in the biosynthesis of a dipeptide (bacilysin), polyketides (difficidin, bacillaene, and macrolactin), and lipopeptides (bacillomycin, fengycin, and surfactin) were identified using PCR in the RDA1 strain. The expected sizes of the obtained PCR products encoding surfactin (*srfAA*) (*bmyB*), fengycin (*fend*), and bacillomycin were 201, 269, and 370 bp, respectively (Figure 6). In addition, the gene *bacA,* involved in the synthesis of the dipeptide bacilysin, as well as the genes involved in the synthesis of the polyketides macrolactin (*mlnA*), bacillaene (*baeA*), and difficidin (*dfnA*) were detected in RDA1 (Figure 6). The results confirmed that RDA1 can produce several distinct types of lipopeptides with antimicrobial properties.

## 3. Discussion

*Bacillus* is a genus of bacteria with a wide range of applications across the industrial, medical, and agricultural fields [14,20,21,22]. Owing to its capacity to control phytopathogens, including *R. necatrix* [23,24], it has attracted attention. *R. necatrix*, a causal agent of white root rot, is a devastating pathogen for which neither curative nor control methods are available. Several approaches have been used to control this pathogen, including soil solarization, soil fumigation [2,25], biological control using the antagonistic rhizobacteria [5,6,24,26], and the fungus *Trichoderma harzianum* [27,28,29]. However, no study has focused on the control of different strains of *R. necatrix* using a single bacterium.

One of the best strategies for selecting microorganisms with antagonistic abilities is the isolation of rhizobacterial strains from the rhizosphere of healthy trees in an area infested with soil-borne phytopathogenic fungi [30]. Here, a new rhizospheric bacterial strain, RDA1 of *Bacillus velezensis*, was isolated from the rhizosphere of a healthy tree in an organic pear orchard infected with *R. necatrix.* The isolated strain was evaluated for its potential antifungal activity against white root rot-causing fungal pathogens and showed a strong inhibitory activity against *R. necatrix*. Several strains of *B. velezensis* isolated from various hosts have demonstrated broad-spectrum antimicrobial and plant growth-promoting properties [31,32]. *B. velezensis* has been used as an antagonist of plant pathogens and a plant growth promoter in sustainable agriculture [4]; for example, the *B. velezensis* strain BS1, isolated from rhizospheric soil in a pepper field, has shown immense potential as a new BCA for controlling pepper anthracnose disease [33]. In this study, the isolated strain RDA1 showed strong antagonistic effects against soil-borne fungal phytopathogens and inhibited the mycelial growth of *R. necatrix* (KACC 40445, KACC 40446, KACC 40447, and KACC 40168). The highest percentage of growth inhibition (81%) was observed in the strain KACC 40447, while the remaining strains also showed a significant inhibition rate of 75–77%. Our observation of different growth inhibition patterns across different strains of *R. necatrix* is consistent with that of a previous study [34] describing that the mycelia of the KACC 40445 strain grew approximately 2–3 times faster in PDA media compared to the 40168 strain. The authors also suggested that *R. necatrix* cell division is primarily regulated at the S-phase cell cycle checkpoints, while the pathogenicity of *R. necatrix* across strains may differ [34]. Therefore, the difference in the pathogenicity of distinct *R. necatrix* strains must be considered when assessing treatments against this pathogen. More comprehensive studies are warranted to better understand the pathogenicity of different *R. necatrix* strains.

The extracellular culture filtrates obtained from RDA1 also inhibited the growth of *R. necatrix,* and this inhibition was positively proportional with the incubation time of CSF collection of RDA1. The highest mycelial growth inhibition was observed against KACC 40445, while the lowest was against KACC 40446. Ohike et al. [35] reported that a 10% culture filtrate of *Bacillus* sp. KL1 inhibited the growth of *Rhizoctonia solani* by 80% on PDA plates, while Xu et al. [36] observed that a culture filtrate of a *Bacillus amyloliquefaciens* strain, LZN01, collected at various incubation points inhibited the growth of *Fusarium oxysporum* by 4–61%. The production of secondary metabolites or antibiotics with a wide array of antimicrobial actions by *Bacillus* species varies with temperature, pH, and growth conditions. These metabolites may include amphipathic cyclic lipopeptides, such as surfactin, iturin, and fengycin, all of which are antifungal agents. We observed that antimicrobial substances secreted by *B. velezensis* RDA1 vary with the growth stage. The amount of antifungal substances secreted varies based on the growth stage.

The antibiotics and VOCs produced by plant-associated antagonistic microbes have received considerable attention [37]. VOCs produced by rhizosphere microbes promote plant growth [38], exhibit nematicidal activity [39], and induce systemic resistance in crops [40]. Yuan et al. [41] have shown that *Bacillus amyloliquefaciens* (NJN-6), isolated from healthy banana plants rhizosphere soil, inhibits *F. oxysporum* spore germination by producing VOCs. These findings indicate that the production of VOCs by microbes associated with plants not only promote plant growth but also provide them a selective advantage over pathogenic microbes by creating a fungistatic microenvironment. In the present study, the biocontrol potential of RDA1 was primarily attributed to its metabolites, such as VOCs and extracellular antibiotics. The VOCs produced by RDA1 inhibited the mycelial growth of all four strains compared to the controls, and the highest percentage of growth inhibition (73.25%) was observed in the strain KACC 40168. Although VOCs have weak antifungal activities compared to nonvolatile antibiotics, they can spread over a long distance, while fungistatic microenvironments develop around the antagonist communities. These results suggest long-distance biocontrol as one mechanism in which RDA1 controls *R. necatrix*.

*Bacillus* species can promote plant growth through several different mechanisms, including the production of siderophores, the synthesis of plant growth-regulating hormones such as IAA, and phosphate solubilization. In the present study, the isolated strain RDA1 possessed plant growth-promoting properties; RDA1 produced IAA and siderophores and exhibited a phosphate solubilization activity. IAA increases both the growth and productivity of plants; PGPB, including *Bacillus* spp., promotes plant growth by producing IAA. Moreover, L-tryptophan, either naturally present in the root exudates or added exogenously, is a precursor for the synthesis of IAA [42]. Exogenous tryptophan supplementation was positively correlated with IAA production in RDA1. IAA production was increased with the supplementation of tryptophan. Likewise, IAA produced by *Bacillus thuringiensis* and *Bacillus cereus* isolated from soil samples increased in the presence of L-tryptophan [43].

Iron (Fe) is an important trace element essential for the growth of microorganisms and plants. However, the oxidized ferric (Fe^3+^) state of Fe, which is largely insoluble at neutral and basic pH levels, reduces its bioavailability in soil despite its abundance. To overcome with iron shortage, plant-associated microbes use ferrireductases to convert Fe^3+^ to Fe^2+^ or solubilize it with extracellular Fe^3+^ chelators known as ‘siderophores’. Siderophores are secondary metabolites that scavenge iron from the environment and deliver it to cells through specific receptors. The water-soluble Fe^3+^-siderophore complexes are readily accessible to microbes and plants. Several *Bacillus* species produce siderophores. Furthermore, siderophores from commensal or mutualistic species can inhibit pathogen growth by depriving them of essential Fe ions [44]. For example, Kesaulya et al. [45] have reported that *Bacillus* sp. isolated from potato rhizosphere soil inhibited the growth of pathogens by producing siderophores.

These reports suggest that the production of siderophores by microbes not only provides plants with access to the limited supply of Fe in the soil but also gives them a selective advantage over pathogenic microbes by blocking pathogens access to iron, which is essential for their survival. The high siderophore production by RDA1 may be partially responsible for its significant antagonistic effects against the tested pathogenic strains. In addition, phosphate solubilization is also one of the most essential characteristics of beneficial bacteria associated with plant growth promotion. In this study, as the only source of phosphate, RDA1 could solubilize inorganic phosphate on a solid NBRIP medium supplemented with Ca_3_(PO_4_)_2_.

RDA1 harbored genes responsible for the biosynthesis of lipopeptides (bacillomycin, fengycin, and surfactin), polyketides (difficidin, bacillaene, and macrolactin), and a dipeptide (bacilysin), which are secondary metabolites produced by PGPB, known for their antimicrobial activities [46,47]. The dipeptide bacilysin is effective against both bacteria and fungi [48]; polyketides are secondary metabolites with antimicrobial activities [48]. Similarly, members of the iturin family lipopeptides (fengycin and bacillomycin D) exhibit potent antibacterial and toxic properties against filamentous and soil-borne fungi [49,50,51,52]. Surfactin is a powerful antifungal biosurfactant [53,54] capable of forming stable biofilms and promoting antagonists colonization in plant tissues [55]. In addition, lipopeptides can induce systemic resistance in the host plants [56]. Collectively, these findings indicate that RDA1 exhibits both plant growth-promoting properties and biocontrol capacity in vitro. Research on field trials, applications, and commercialization is currently being conducted.

## 4. Materials and Methods

### 4.1. Pathogens

Four isolates of *Rosellinia necatrix* (KACC 40445, 40446, 40447, and 40168) were acquired from Korean Agricultural Culture Collection (KACC, National Institute of Agricultural Sciences, Iseo-myeon, Wanju_Gun, 55365, Jeollabuk-do, Korea). The isolates were maintained on a potato dextrose agar (PDA) medium at 25 ± 2 °C in the dark and stored at 4 °C.

### 4.2. Soil Sampling

The rhizosphere of healthy trees in areas infested with soil-borne phytopathogenic fungi is a feasible and useful source for the isolation of microorganisms with potential antagonistic abilities [30]. Soil samples from organic pear orchards infested with white root rot are a good source of antagonistic bacteria. Eight rhizosphere soil samples were collected from a pear tree in organic orchards located around Naju City (Jeollanm-do Province, South Korea). The rhizosphere soil of the pear tree was dug, and the root portion of the soil was placed in a polythene bag, labeled, tied, and placed in an icebox. The icebox was quickly to the laboratory and stored at 4 °C until use.

### 4.3. Isolation of Bacterial Strains

A mixed rhizosphere soil sample was collected from the roots and surrounding areas, as described in the soil sampling. One gram of soil sample was mixed with 10 mL of sterile phosphate-buffered saline (PBS; pH 7.2). The saline soil samples were diluted serially (up to 10^-6^-fold), spread on PDA plates, and incubated at 28 ± 2 °C for 2–3 days. Bacterial colonies that appeared on the plates after incubation were selected and isolated based on their visual characteristics and preserved on PDA slants at 4 °C for later studies.

### 4.4. Screening and Selection of Antagonistic Bacteria

All bacterial isolates were initially screened for antifungal activity against the white root rot pathogen *R. necatrix* using the dual-culture method with a slight modification. Briefly, a 10-day-old mycelial plug of *R. necatrix* (5 mm) obtained from the PDA culture was placed at the center of a fresh PDA plate in a 90 mm Petri dish and incubated in the dark for 24 h at 25–27 °C. On a PDA plate, a bacterial sample was streaked 2.0 cm away from the fungal pathogen and incubated in the dark for 10 days at 25–27 °C. Several isolates exhibited antagonistic activity against *R. necatrix*. Among these, the most effective antagonistic bacterial strain (RDA1) with high inhibitory activity (70–80%) against all strains of *R. necatrix* (KACC 40168, 40445, 40446, and 40447) was selected for further characterization.

### 4.5. 16S rRNA Gene Analysis

RDA1 was selected for further characterization. The pure culture was sequenced by the commercial sequencing company Macrogen (Seoul, Korea). The sequence of the isolate in this study was deposited in GenBank under accession number ON159294. The 16S rRNA gene sequence was compared to all available sequences in databases using the Basic Local Alignment Search Tool (BLAST) server at the National Center for Biotechnology Information (NCBI). The sequence was aligned with the reference strains using CLUSTAL W [57]. The neighbor-joining method [58] was used to create a phylogenetic tree using MEGA X software (https://www.megasoftware.net/, accessed on 18 April 2022). Bootstrap percentages were calculated using 1000 replicates.

### 4.6. Antifungal Activity Assays

The antifungal activity of RDA1 was evaluated using a dual-culture plate assay as described by Anith et al. [59]. Each bacterial isolate had two bands (3 cm long) streaked parallel to each other on PDA plates at a distance of 3 cm from the center of the plates and incubated at 25 °C for 24 h. A mycelial disc with a diameter of 5 mm was cut from the growing edge of a 5-day-old colony of the phytopathogen *R. necatrix* (KACC 40446, 40445, 40447, and 40168) using a sterile cork borer and placed at the center of the PDA plate inoculated with the bacterial isolate. PDA plates inoculated with *R. necatrix* alone were used as the controls. Plates inoculated with an antagonistic bacterial strain and phytopathogen (test plates) and phytopathogen alone (control plates) were incubated for 10 days, and the radii of the fungal colonies (R1 and R2) in the test plates and those (CR1 and CR2) in the control plates were measured. For each antagonist–phytopathogen combination, test and control plates were prepared. The experiment was repeated three times with three replicates. The inhibitory effect of the bacterial isolates was calculated as an inhibition percentage (I%).

I(%) = (CR − R)/CR × 100

where CR (mean of CR1 and CR2) is the radius of the fungal colony in the control plates and R (mean of R1 and R2) is the radius of the fungal colony in the test plates.

### 4.7. Antagonistic Efficacy of the Cell-Free Supernatant (CFS)

The antifungal activity of RDA1 CFS was determined by calculating the percentage of mycelial growth inhibition of *R. necatrix* (KACC 40446, 40445, 40447, and 40168) on PDA plates. Briefly, to assess the effect of the CFS collected at various incubation points on *R. necatrix* mycelial growth, the RDA1 strain was grown in 250 mL Erlenmeyer flasks separately according to the CFS collection time point (24, 36, 48, 60, 72, and 90 h) containing 100 mL of LB (Luria-Bertani) medium at 30 °C. The extracellular filtrate was collected via centrifugation at 16,000× *g* at 4 °C for 10 min and sterilized using a 0.22 µm pore diameter filter. PDA was mixed with the sterilized extracellular filtrates (10% *v*/*v*). Subsequently, 10 mL aliquots of the mixture were poured into Petri dishes (60 mm in diameter). To prepare control plates, sterilized LB media was mixed with PDA. A 4 mm fungal block was placed in the center of each plate and incubated at 28 °C for 10 days, following which the mycelial growth inhibition was calculated as follows:

I = (C − T)/C × 100

where I (%) is the mycelial growth inhibition, C (mm) is the mycelial diameter in the control, and T (mm) is the mycelial diameter in PDA containing RDA1 supernatant [35,60].

### 4.8. Antagonistic Assay of Volatile Organic Compounds (VOCs) against Fungi

The antifungal activity of the VOCs produced by RDA1 was assessed after culturing on two sealed Petri dishes [61]. One Petri dish contained 10 mL of LB medium, while the other plate contained 10 mL of PDA medium. A 4 mm diameter plug of *R. necatrix* (KACC 40445, 40446, 40447, and 40168) was placed on the PDA plate, and 50 μL of RDA1 suspension was spread on the LB medium plate. To prevent the loss of volatiles produced, the two culture plates were placed facing each other and sealed together using a double layer of paraffin film. As a positive control, LB plates with no bacterial cultures were covered and sealed with *R. necatrix*-inoculated PDA plates. Pathogen growth was measured after 10 days of incubation at 27 °C. Each experiment was performed in triplicate. Fungal growth inhibition (I) was calculated as follows:

I = [(C − T)/C] × 100

where C is mycelial growth in the control and T is mycelial growth in the treatment.

### 4.9. Measurement of PGP Activities of the RDA1 Isolate

Crucial plant growth-promoting traits such as phosphate solubilization, and IAA and siderophore production of RDA1 were analyzed. Phosphate solubilization was assessed using the method developed by Mehta and Nautiyal [62]. Briefly, RDA1 was grown in a solid National Botanical Research Institute’s phosphate growth medium NBRIP (10 g/L glucose, 5 g/L Ca_3_(PO_4_)_2_, 5 g/L MgCl_2_, 0.1 g/L (NH_4_)_2_SO_4_, 0.25 g/L MgSO_4_·7H_2_O, 0.2 g/L KCl, and 15 g/L agar), and its use of Ca_3_(PO_4_)_2_ as the sole phosphate source was assessed. Plates were incubated for 14 days at 28 °C, following which the formation of a halo around the colony indicated phosphate solubilization. The halo diameter directly correlated with phosphate solubilization. IAA production in the isolated strain RDA1 was measured as previously described [19]. Isolated colonies were inoculated into Jensen’s broth (sucrose 20 g/L, K_2_HPO_4_ 1 g/L, MgSO_4_ 7H_2_O 0.5 g/L, NaCl 0.5 g/L, FeSO_4_ 0.1 g/L, NaMoO_4_ 0.005 g/L, and CaCO_3_ 2 g/L) supplemented with or without 2 mg ml^−1^ L-tryptophan. The culture was incubated for 48 h at 28 ± 2 °C with continuous shaking at 125 rpm. After incubation, a 2 mL culture solution was collected and centrifuged at 14,000 rpm for 1 min, and 1 mL of the supernatant was mixed with 2 mL of the Salkowski’s reagent (1 mL of 0.5 M FeCl_3_ solution in 50 mL of 35% perchloric acid) and incubated in the dark for 20 min at 25 °C. The appearance of a pink-red color indicates IAA production. A standard curve prepared from pure IAA solutions (0, 10, 20, 30, 40, 50, and 60 μg mL^−1^) was used to determine IAA concentration. Absorbance at 540 nm was measured using a spectrophotometer (BioTek instruments, Winooski, VT, USA).

For the quantitative estimation of siderophores, a Chromium Azurol S (CAS) assay was performed as previously described [19]. Briefly, bacterial cells were cultured in a modified M9 liquid medium lacking Fe and incubated for 2 days with constant shaking. After incubation, 10 mL of culture was collected and centrifuged for 5 min at 15,000 rpm. The supernatant was collected and filtered through a 0.2 μm membrane filter. The filtrate (0.5 mL) was mixed with the CAS assay solution (0.5 mL) and incubated for 3–4 h. The presence of siderophores was confirmed by a change in color from blue to orange. The quantity of siderophore produced was determined as follows:

%Siderophore unit = [(Ar − As)/Ar] × 100

where Ar is the absorbance of reference (CAS reagent) and As is the absorbance of the sample at 630 nm. All assays were performed in triplicates.

### 4.10. Detection of Antibiotic Biosynthesis Genes

A polymerase chain reaction (PCR) was performed to determine the genes involved in the synthesis of lipopeptide, polyketide, and dipeptide. The primers used for the detection of antibiotic biosynthesis genes are listed in Table 2. The reaction mixture (total volume of 20 µL) contained 10 µL of the master mix of dNTPs, Taq DNA polymerase, and MgCl2, 1 µL of each forward and reverse primer (10 mmol/L), and 100 ng of template DNA.

The PCR cycling conditions were as follows: initial denaturation at 95 °C for 5 min, 30 cycles of denaturation at 95 °C for 1 min, 50–58 °C primer annealing for 1 min, extension at 72 °C for 1.5 min, and a final extension step at 72 °C for 7 min.

### 4.11. Statistical Analysis

Data were analyzed using SAS (SAS Institute Inc., Cary, NC, USA) program. The data were subjected to one-way analysis of variance (ANOVA) followed by Duncan’s multiple range test, with a significance level of *p*  <  0.05.

## 5. Conclusions

The isolated *B. velezensis* strain RDA1 exhibited strong antagonistic effects against different strains of *R. necatrix*. This potentially high antifungal activity may be attributed to the bioactive compounds previously identified as antimicrobials in nature. CFS and VOCs of RDA1 exhibited strong antifungal activity against *R. necatrix*. Moreover, RDA1 exhibited several plant growth-promoting traits. Therefore, RDA1 may be a good source for BCAs against white root rot and as a bio-fertilizer in sustainable agriculture.

## Figures and Tables

**Table 1:** Plant growth-promoting characteristics of RDA1.

PS	IAA Production in the Presence of Tryptophan	IAA Production in the Absence of Tryptophan	Siderophore (PSU)
+++	73.6 ± 4.9	23.2 ± 1.9	82.6 ± 4.3

Results are presented as the mean ± standard deviation of three independent experiments, with each treatment measured three times. PS, phosphate solubilization; IAA, indole acetic acid. Evaluation of the positivity of the tests: (+++) indicates highest, activity.

**Table 2:** The list of primers used to detect the genes involved in the biosynthesis of lipopeptides, polyketides, and dipeptides.

Lipopeptides	Gene	Primer	Sequence (5′-3′)	Annealing Temperature (°C)	PCR Product Size (bp)	Reference
Bacillomycin	*bmyB*	BMYB-F	GAATCCCGTTGTTCTCCAAA	55	370	[50]
		BMYB-R	GCGGGTATTGAATGCTTGTT			
Fengycin	*fend*	FEND-F	GGCCCGTTCTCTAAATCCAT	58	269	[50]
		FEND-R	GTCATGCTGACGAGAGCAAA			
Surfactin	*srfAA*	SRFA-F	TCGGGACAGGAAGACATCAT	58	201	[50]
		SRFA-R	CCACTCAAACGGATAATCCTGA			
Macrolactin	*mlnA*	mlnA-F	CCGTGATCGGACTGGATGAG	58	668	[63]
		mlnA-R	CATCGCACCTGCCAAATACG			
Bacillaene	*baeA*	baeA-F	ATGTCAGCTCAGTTTCCGCA	59	688	[63]
		baeA-R	GATCGCCGTCTTCAATTGCC			
Difficidin	*dfnA*	dfnA-F	GGATTCAGGAGGCATACCG	59	653	[63]
		dfnA-R	ATTGATTAAACGCGCCGAGC			
Bacilysin	*BacA*	BacA-F	CAGCTCATGGGAATGCTTTT	58	498	[50]
		BacA-R	CTCGGTCCTGAAGGGACAAG			

## Data Availability

The sequence for the isolate in this study was deposited in GenBank under accession number ON159294.
